# Structural and Electrochemical Properties of Physically and Chemically Activated Carbon Nanoparticles for Supercapacitors

**DOI:** 10.3390/nano12010122

**Published:** 2021-12-30

**Authors:** Nuha A. Alhebshi, Numan Salah, Humair Hussain, Yousef N. Salah, Jian Yin

**Affiliations:** 1 Physics Department, Faculty of Science, King Abdulaziz University, Jeddah 21589, Saudi Arabia; humair004@gmail.com; 2 Centre of Nanotechnology, King Abdulaziz University, Jeddah 21589, Saudi Arabia; nsalah@kau.edu.sa; 3 Department of Chemical and Materials Engineering, Faculty of Engineering, King Abdulaziz University, Rabigh 21911, Saudi Arabia; ynsalah@gmail.com; 4 Materials Science and Engineering, Physical Science and Engineering Division, King Abdullah University of Science and Technology (KAUST), Thuwal 23955-6900, Saudi Arabia; jian.yin@kaust.edu.sa

**Keywords:** date palm, activated carbon, chemical activation, physical activation, nanostructure, specific surface area, supercapacitor, specific capacitance

## Abstract

The demand for supercapacitors has been high during the integration of renewable energy devices into the electrical grid. Although activated carbon materials have been widely utilized as supercapacitor electrodes, the need for economic and sustainable processes to extract and activate carbon nanomaterials is still crucial. In this work, the biomass waste of date palm fronds is converted to a hierarchical porous nanostructure of activated carbon using simple ball-milling and sonication methods. Chemical and physical activation agents of NaOH and CO_2_, receptively, were applied on two samples separately. Compared with the specific surface area of 603.5 m^2^/g for the CO_2_-activated carbon, the NaOH-activated carbon shows a higher specific surface area of 1011 m^2^/g with a finer nanostructure. Their structural and electrochemical properties are functionalized to enhance electrode–electrolyte contact, ion diffusion, charge accumulation, and redox reactions. Consequently, when used as electrodes in an H_2_SO_4_ electrolyte for supercapacitors, the NaOH-activated carbon exhibits an almost two-fold higher specific capacitance (125.9 vs. 56.8 F/g) than that of the CO_2_-activated carbon at the same current density of 1 A/g. Moreover, using carbon cloth as a current collector provides mechanical flexibility to our electrodes. Our practical approach produces cost-effective, eco-friendly, and flexible activated carbon electrodes with a hierarchical porous nanostructure for supercapacitor applications.

## 1. Introduction

As the share of renewable energy increases in the electric grid, supercapacitors play an increasingly crucial role in its integration [1,2]. Supercapacitors have the merits of fast operation rates, high power densities, ultralong cycle lifes, and low energy densities [3]. Based on the charging mechanism of the electric double-layer capacitor, the specific surface area of carbon electrodes needs to be as high as possible to theoretically achieve high capacitance [4,5]. However, because of the poor compatibilities between microporous structures and electrolyte ions, porous carbons always show limited capacitances and hysteretic current responses [6,7]. Hierarchical porous carbons, such as activated carbon (AC) and carbon nanotubes (CNTs) enable more advanced electrolyte transportation than microporous carbons [8,9,10]. When used in supercapacitors, macropores act as tanks for electrolyte storage, mesopores offer fast ion transportation channels, while micropores provide multiple active sites for electric double-layer capacitance [11,12]. Nevertheless, the fabrication of macropores and mesopores usually relies on templates. Such variety in the porosity and electrical conductivity of carbon materials enable supercapacitors to be compatible with a wide range of energy- and power-demand applications. For example, AC-based electrodes stored energy at higher densities but with lower power densities than AC-CNT-based electrodes as compared by C. Lei and C. Lekakou [13]. Focusing on AC materials, different types of commercial AC were used in individual supercapacitors offering either high power or high energy densities. Then, they were combined into composite supercapacitors designed and tested in medium- and large-scale systems [14]. To effectively utilize the porosity of the electrode materials, the size of electrolyte ions should be matched with the pore size, as theoretically and experimentally investigated in aqueous and non-aqueous electrolytes [15,16,17]. In supercapacitors with 1 M H_2_SO_4_ aqueous electrolyte, a disordered micro–mesoporous AC electrode revealed a specific capacitance of 156 F/g at a current density of 0.5 A/g with an energy density of 7.8 Wh/kg [17], while a microporous AC electrode with an average pore size of 1.96 nm displayed 247 F/g at 0.5 A/g and 101.1 Wh/kg [18]. These studies practically confirm that the micropores can be suitable sites for the hydrated ions of H_2_SO_4_.

Biomass materials possess a multistage channel structure for nutrient transport. Biomass-derived carbon inherits the macroporous and mesoporous structures of tissue naturally [19,20]. With a common activation procedure, biomass-derived activated carbons usually show hierarchical structures rich in macro, meso, and micropores [21,22,23]. Thus, biomass-derived activated carbons can deliver high capacitances and excellent rate performances. The date palm has been grown and cultivated as a crop in the MENA region for hundreds of years [24]. Considering that a large quantity of biomass waste is produced by date palms every year, the conversion of biomass waste into valuable activated carbons provides functional electrode materials. Importantly, it also addresses environmental issues such as cost-effectiveness and sustainability in energy development [24,25,26].

Researchers have been looking for cost-effective methods to develop activated carbons using the biomass waste produced by date palms [27]. Alhamed et al. used date pits as the carbon precursor and ZnCl_2_ as the activation agent to prepare activated carbon [28]. The activated carbon at 600 °C with an activation agent ratio of 0.5 for 3 h shows a specific surface area of 999 m^2^/g and a pore volume of 0.53 cm^3^/g. The N_2_ adsorption and desorption isotherms indicated that the activated porous carbon has a wide pore size distribution. Reddy et al. investigated the influence of the activation agents H_3_PO_4_ and CO_2_ on the porous structure of activated carbons prepared using date pits as the carbon precursor [29,30]. The CO_2_-activated carbon shows a microporous structure with a specific surface area of 666 m^2^/g, while the H_3_PO_4_-activated carbon shows a mesoporous structure with a specific surface area of 725 m^2^/g. Merzougui et al. prepared activated carbon using date pits with the activation agents ZnCl_2_ and KOH [31]. The carbon prepared by the direct carbonization shows a specific surface area of 640 m^2^/g and a pore volume of 0.65 cm^3^/g. After the activation by ZnCl_2_ and KOH, the specific surface area increased to 882 and 1032 m^2^/g with pore volumes of 1.06 and 1.21 cm^3^/g, respectively. Accordingly, although biomass-derived carbon inherits a partially porous structure from the channels used for the nutrient supply system, the activation agents affect activated carbons’ specific surface area and porous structure. Therefore, more investigation on the activation process is needed to develop activated carbon materials for high-performance supercapacitors.

In the activation process, the most widely used chemical activation agents are KOH, NaOH, H_3_PO_4_, and ZnCl_2_, and the most commonly used physical activation agents include CO_2_ and steam, respectively [32,33,34]. Generally, KOH is a chemical activation agent that can develop a high specific surface area and micropore volume but incurs a high cost [35,36]. Compared with the other activation agents, NaOH and CO_2_ can create a relatively higher specific surface area and lower the fabrication cost [37,38,39]. Thus, the activation processes using NaOH and CO_2_ are cost-effective and beneficial for the large-scale production of activated carbon materials.

For supercapacitor applications, chemically and physically activated carbons, respectively denoted C-AC and P-AC, were tested in different types of electrolytes. For example, a C-AC electrode from an oil palm kernel shell delivered a specific capacitance of 74.7 F/g in H_2_SO_4_, 58.8 F/g in KOH, and 52.8 F/g in Na_2_SO_4_ [40]. In the same electrolytes, P-AC offered 74.2 F/g, 71.5 F/g, and 44.5 F/g, respectively, measured at a current density of 0.3 A/g. Another study concluded that oxygen in AC contributes to reduction and oxidation (redox) reactions, boosting the specific capacitance in the acidic electrolyte more effectively than in the basic electrolyte [41]. Based on that, the acidic electrolyte seems to be preferred for both the chemically and physically activated carbons.

Herein, we utilize date palm fronds to produce cost-effective, eco-friendly, and flexible activated carbon electrodes with a hierarchical porous nanostructure using ball-milling and sonication. The chemical and physical activation agents NaOH and CO_2_ were applied on two samples separately. Their structural and electrochemical properties are investigated in terms of specific surface areas, porosities, redox activities, electrochemical impedances, and specific capacitances. Interestingly, a two-fold improvement in specific capacitance for our supercapacitor electrodes is achieved and analyzed.

## 2. Materials and Methods

### 2.1. Materials Synthesis and Activation

The carbon nanostructures produced from carbonized date palm fronds were synthesized using the high-energy ball-milling and sonication techniques as described in our previous USA patent [42]. In this method, the particle size of 10 g of the carbonized sample was reduced by putting it in ceramic jars along with the corresponding balls of the high-energy ball-milling technique. Five to seven hours is enough to reduce the particle sizes into the nanoscale. The sonication process was followed by taking a few grams of the ball-milled sample and keeping it in a water medium (50–100 mL). The sample was sonicated for 3 h using a high-power ultrasonic probe at 200 W. Finally, the resulting ultrafine powder was collected by drying the sonicated sample in an oven at 80 °C and was used for further analysis. Physical and chemical methods activated the produced samples. The physical activation was performed in a vacuum furnace at 900 °C for 90 min under CO_2_ gas flow at a pressure of 320 Torr. In the chemical activation, the carbonized nanoparticles were impregnated with NaOH at a ratio of 1:4 by weight in a water medium. The solution was stirred for 2 h; then, the impregnated sample was dried at 100 °C for several hours. The dried material was placed in a vacuum furnace in an open crucible under a nitrogen gas flow at 700 °C for 2 h. Finally, the sample was washed with DI water and acid until it reached neutral pH.

### 2.2. Materials Characterization

The morphology of the activated carbon samples was imaged at the micro and nanoscales by scanning electron microscope (SEM JSM-7500F, JEOL, Tokyo, Japan). The SEM images were generated using an accelerated electron beam at 15 kV and detected by the secondary electron detector (SE). The pore structure was characterized by N_2_ adsorption–desorption at a temperature of −196.15 °C (77 K) via the surface area and pore size analyzer (Nova 1200e, Quantachrome Instruments, Boynton Beach, FL, USA). From the adsorption–desorption isotherm data, the Brunauer–Emmett–Teller (BET), density functional theory (DFT), and Dubinin–Astakhov (DA) methods were applied to calculate the specific surface area and porosity of the activated carbon. The chemical surface functionalization and purity were determined using an X-ray photoelectron spectroscope (XPS PHI 5000 VersaProbe, PHI, Chigasaki, Japan). A complete survey and narrow spectrum (from 292 to 280 eV) were recorded. Furthermore, a Raman microscope (DXR, Thermo Fisher Scientific, Waltham, MA, USA) was operated with a laser wavelength of 532 nm to confirm the presence of hybridized graphitic orbitals.

### 2.3. Electrodes Testing

To fabricate carbon-based electrodes, our activated carbon was mixed with polytetrafluoroethylene (PTFE) as a binder in a ratio of 80:20 in ethanol using sonication. Then, the mixture was dropped onto several 1 cm^2^ pieces of carbon cloth followed by drying in a box furnace at 60 °C. The carbon substrates were weighed before and after coating to determine the electrodes’ mass loadings, which were found to be 1.4 mg/cm^2^ and 1.8 mg/cm^2^, respectively, for NaOH-activated and CO_2_-activated carbon electrodes. These electrodes were considered the working electrodes in a standard configuration of a three-electrode system, with a reference electrode of silver/silver chloride (Ag/AgCl) and a 1 cm^2^ platinum sheet counter electrode. The electrochemical workstation (CHI 660D Model, CH Instruments Incorporation, Austin, TX, USA) was used to conduct the following electrochemical tests at room temperature with 1 M H_2_SO_4_ being used as an aqueous electrolyte. Cyclic voltammetry (CV) experiments were carried out at scan rates between 10 mV/s and 500 mV/s within a potential window of 1 V to identify the charge storage mechanism. In addition, the specific (gravimetric) capacitances of the electrodes were calculated at current densities from 0.4 A/g to 20 A/g by galvanostatic charge–discharge (GCD) curves according to the equation:

(1) $$C_{s}=\frac{I\;\Delta t}{m\;\Delta V}$$

where I/m is the applied current density in amperes per gram (A/g), ∆t is the time of the discharge curve in seconds (s), and ∆V is the potential window in volts (V). Besides this, Nyquist plots were generated from the electrochemical impedance spectroscopy (EIS) measurements in a voltage amplitude of 5 mV with a frequency range of 100 kHz–0.1 Hz. The complex impedance diagram represents the equivalent series resistance (ESR), charge transfer resistance (R_CT_), and electrolyte diffusion into the electrode.

## 3. Results and Discussion

### 3.1. Materials Properties

**Figure 1:**
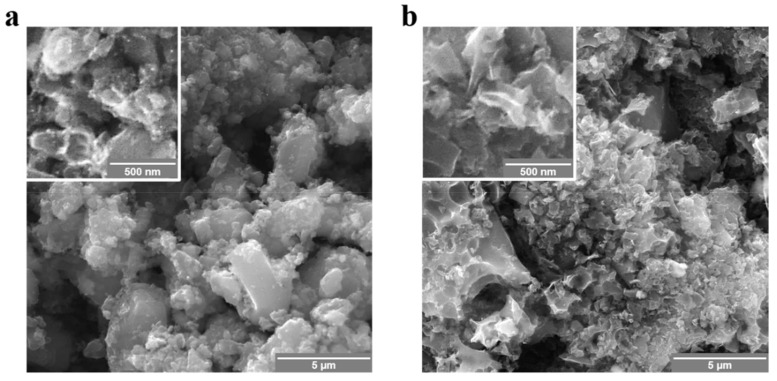
SEM images at different magnifications of (**a**) the physically CO_2_-activated carbon and (**b**) the chemically NaOH-activated carbon.

Figure 1 shows SEM images of the as-prepared samples at nanometer and micrometer scales. The carbon microparticles are shattered into smaller pieces in nonuniform shapes using ball-milling at high energy, while the sonication process contributes to distributing the milled particles homogenously. Although clusters of microparticles and nanostructured particles can be seen in both samples, the chemically NaOH-activated carbon seems to have better a morphology in terms of the finer nanosheets present compared to the carbon that was activated physically by CO_2_.

**Figure 2:**
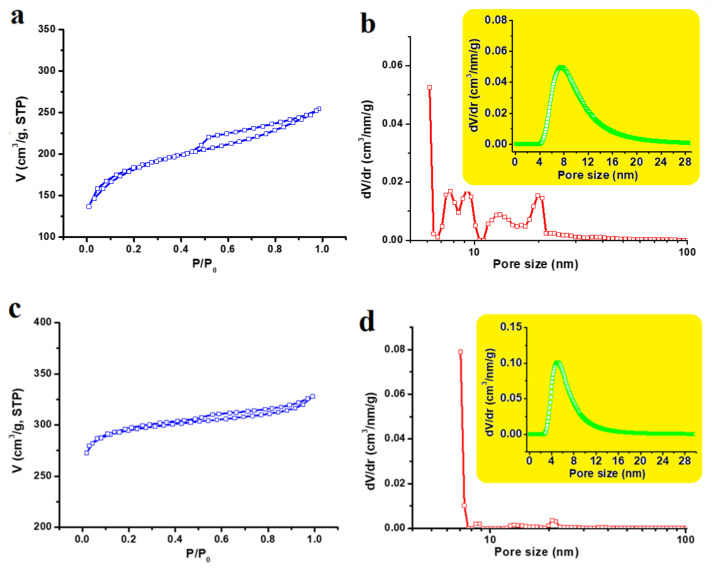
N_2_ adsorption–desorption isotherms and pore size distributions calculated by DFT; (**a**,**b**) for physically CO_2_-activated carbon; (**c**,**d**) for chemically NaOH-activated carbon; The insets are the pore size distribution calculated by the DA method.

The activation step is crucial for the carbon to generate the porous structure and increase the specific surface area. As calculated from Figure 2a,c, the BET surface area for the physically activated sample is 603.5 m^2^/g, while that of the chemically activated sample is 1011 m^2^/g. These values are compatible with the reported 666 m^2^/g for the CO_2_-activated carbon [30], and 1032 m^2^/g for the KOH activated carbon [31]; both were derived from date pits. As represented in Figure 2b,d, they are microporous within the ranges 4–20 and 4–12 nm in diameter, respectively, in the physically and chemically activated samples. In addition, Table 1 lists all values of the micropore volumes in units of cm^3^/g as calculated by the DFT and DA methods. Such porosity in supercapacitor electrodes plays a crucial role in reinforcing the electric double-layer formation, where the pores can be accessible to electrolyte ions.

**Figure 3:**
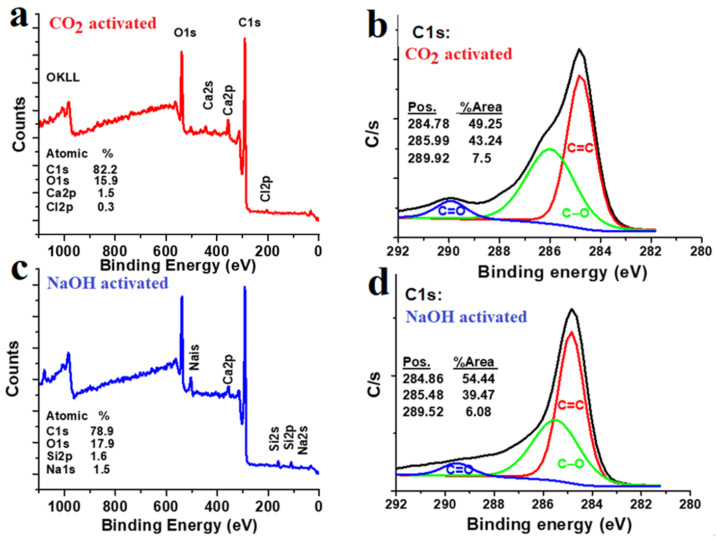
XPS full-survey and C 1s profiles; (**a**,**b**) for the physically CO_2_-activated carbon; (**c**,**d**) the chemically NaOH-activated carbon.

The XPS spectra of the physically CO_2_- and chemically NaOH-activated carbon are analyzed in Figure 3. The complete survey profiles of both samples confirm that carbon (C 1s) is the primary element with a considerable amount of oxygen (O 1s). Such oxygen could contribute to charge storage, as examined in the next section of electrode performance. The percentages of the impurities are less than 1.7% for each element of calcium (Ca 2p) and chloride (Cl 1s) in the first sample, and silicon (Si 2p) and sodium (Na 1s) in the second sample. These negligible traces prove the power of the sonication and washing steps. The C 1s profile of the physically CO_2_-activated carbon is deconvoluted into three peaks positioned at binding energies of 284.78 eV, 285.99 eV, and 289.92 eV, corresponding to C=C, C-O, and C=O chemical bonds, respectively [43,44]. In the chemically NaOH-activated carbon, the peaks are centered at 284.86 eV, 285.48 eV, and 289.52 eV, and represent the same types of carbon and oxygen bonds. The oxygen-containing functional group is successfully attached to the surface of the carbon.

**Figure 4:**
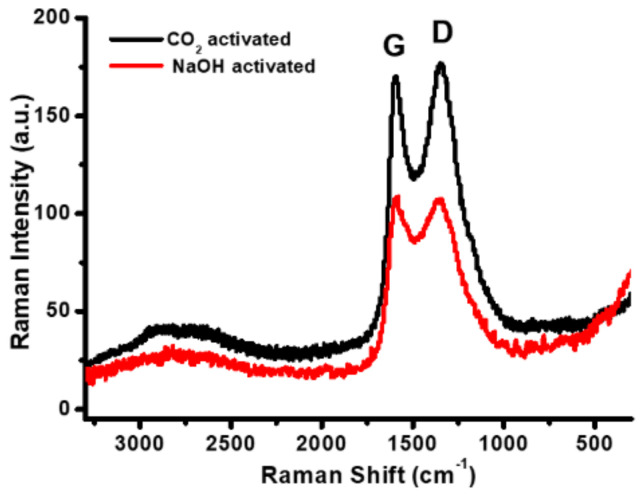
Raman spectra of the physically CO_2_- and chemically NaOH-activated carbon.

For further determination of the carbon bonds, Raman spectra of both samples are compared in Figure 4. There are obvious G-band and D-band peaks generated by the in-plane vibrational modes of the carbon bonds. In particular, the double bond (C=C) of the hybridized sp^2^ orbital in the graphitic ordered layers, as indicated by the G-band. In contrast, the graphitic disordered layers are confirmed by the D-band [45]. The D and G peak intestines (I_D_/I_G_) ratios are close to 1 in both samples, consistent with the value reported for many standard activated carbon materials [46]. As a result, each of the physically and chemically activated carbon samples in our study is known to have a combination of amorphous and crystalline structures. The presence of amorphous material is recommended for supercapacitor electrodes due to its porosity and vacancies [47,48].

### 3.2. Electrodes Performance

**Figure 5:**
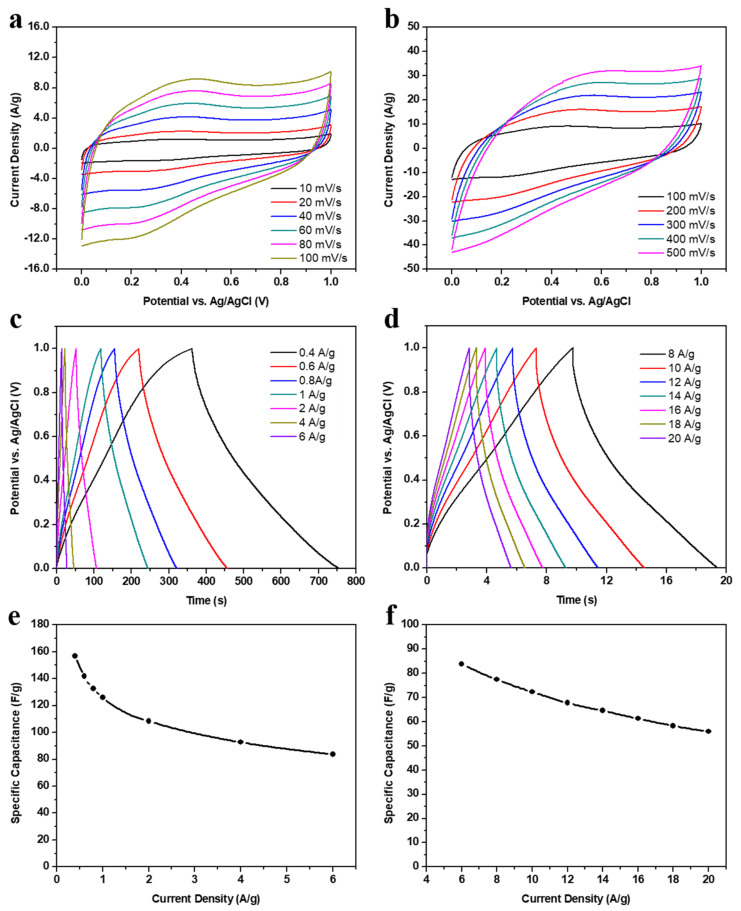
The electrochemical performance of the chemically NaOH-activated carbon electrode; (**a**,**b**) cyclic voltammetry curves at low and high scan rates, respectively; (**c**,**d**) galvanostatic charging–discharging plots at low and high current densities, respectively; (**e**,**f**) specific capacitance functions at low and high current densities, respectively.

The electrochemical energy storage mechanisms of our NaOH-activated carbon electrode in 1 M H_2_SO_4_ are investigated by the CV curves in Figure 5a,b. In a potential window from 0.0 V to 1.0 V vs. SCE, the anodic and cathodic current density functions demonstrate a semi-rectangular shape with broad peaks, representing the electric double-layer formation and the Faradaic redox reactions. These reversible processes are effectively facilitated at the electrode–electrolyte interface due to the microporous structure and the large BET-specific surface area (1011 m^2^/g). It has been proved that matching the electrode porosity to the hydrated electrolyte ions is a fundamental feature in electric double-layer capacitors, while attaching oxygen-containing functional groups to the carbon surface provides a valuable pseudocapacitive contribution [49]. In particular, the carbon–oxygen bonds induce redox reactions with the acidic aqueous electrolyte [41]. Notably, the potential separation between the current peaks of our samples is nearly fixed, indicating that the redox reactions have good stabilities at different scan rates, particularly from 10 mV/s to 100 mV/s.

By applying a wide range of current densities, the GCD curves of Figure 5c,d are generated within 1 V. The expected semi-triangular plots with small plateaus confirm the charge storage mechanisms mentioned above. It can be seen that the electrode can be charged and discharged slower at lower current densities than at higher values. Applying high current densities could accelerate the charge accumulation on the electrode surface, hence electrostatically attracting the opposite electrolyte ions to build the electric double-layer before completing the redox reactions sufficiently. Such chemical reactions require a longer time at lower current densities to effectively contribute to charge storage. This well-known inversely proportional relationship results in an increase of the specific capacitance of our NaOH-activated carbon electrode from 55.9 F/g at 20 A/g to 156.8 F/g at 0.4 A/g, as calculated by the Equation (1) and represented in Figure 5e,f. A similar value of 150 F/g at 0.3 A/g was reported by Farma et al. using KOH-CO_2_-activated carbon from empty fruit bunches of oil palm on stainless steel foil in 1 M H_2_SO_4_ [50]. In another example, a commercial activated carbon was treated and added to waste palm and activated with Na_2_ and KOH in a microwave-heating process. Then, the resultant porous carbon was mixed with acetylene black and PTFE on a piece of nickel foam and exhibited 226.0 F/g at 0.5 A/g in a gel electrolyte of polyvinyl alcohol (PVA) and lithium chloride (LiCl) [51]. By comparison, it can be noted that our electrode has excellent performance without a need for commercially activated carbon or conductive additives. Moreover, using carbon cloth as a woven current collector instead of stainless-steel foil provides flexibility to our electrodes.

**Figure 6:**
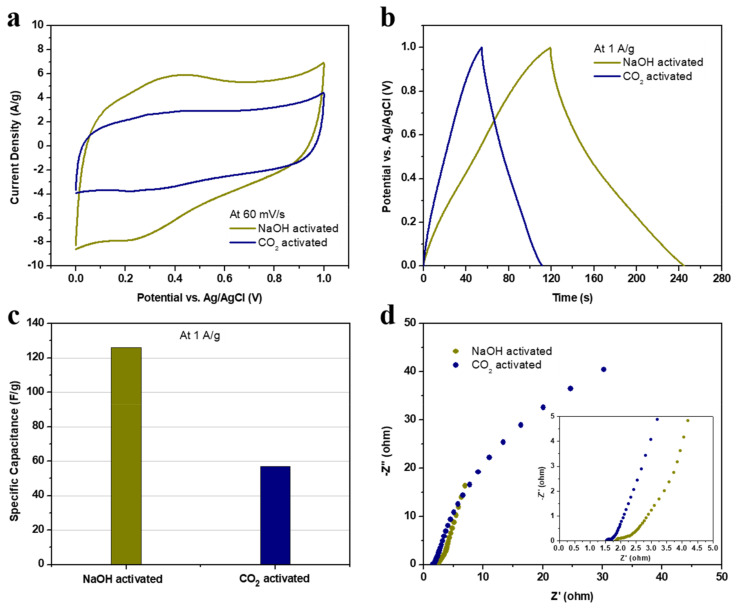
The electrochemical performance of the physically CO_2_- and chemically NaOH-activated carbon electrodes in terms of (**a**) a cyclic voltammetry curve at 60 mV/s, (**b**) a galvanostatic charging–discharging plot at 1 A/g, (**c**) specific capacitance values at 1 A/g, and (**d**) a Nyquist plot of electrochemical impedance spectroscopy with the high-frequency region in the inset.

Figure 6 compares the electrochemical results between the NaOH- and CO_2_-activated carbon electrodes. At a scan rate of 60 mV/s, the mathematical area under the semi-rectangular CV curve of the NaOH-activated electrode is more significant than that of the CO_2_-activated electrode. Similarly, the charge–discharge time at 1 A/g for the first electrode is approximately double the discharge time of the second electrode. Therefore, NaOH-activated electrode exhibits an almost two-fold higher specific capacitance than that of the CO_2_-activated electrode at the same current density of 1 A/g (125.9 vs. 56.8 F/g). Besides this, the electrolyte ions diffuse into the later electrode material with higher electrochemical impedance, as pointed out in the Nyquist plot at low frequencies. At high frequencies on the other hand, the ESR of the NaOH-activated electrode is 1.842 ohm which is higher than the 1.544 ohm of the other electrode due to the difference in oxygen content of our electrodes, which is 17.9% vs. 15.9%, respectively. The apparent improvement in the electrode performance is ascribed to the synthetic conditions and material properties analyzed in the previous sections. In a nutshell, activating the carbon chemically by NaOH produces finer particles, smaller pores, a larger BET specific area, and higher oxygen atomic percentages than those of the physically CO_2_-activated carbon. These optimized properties are functionalized when used in supercapacitors to enhance the electrode–electrolyte contact, ion diffusion, charge accumulation, and redox reactions.

## 4. Conclusions

In this work, the biomass waste of date palm fronds was converted to carbon nanostructured material for use as supercapacitor electrodes. Ball-milling and sonication were used to generate fine micro and nanoparticles, which are beneficial for generating full contact with the activation agents during the activation process. The chemical and physical activation agents NaOH and CO_2_ were applied to two samples separately. Compared with the specific surface area of 603.5 m^2^/g for the CO_2_-activated carbon, the NaOH-activated carbon shows a higher specific surface area of 1011 m^2^/g and a hierarchical porous nanostructure with a wide pore size distribution. Consequently, when used as electrodes in an H_2_SO_4_ electrolyte for supercapacitors, the NaOH-activated carbon exhibits an almost two-fold higher specific capacitance (125.9 vs. 56.8 F/g) than that of the CO_2_-activated carbon at the same current density of 1 A/g. The structural and electrochemical properties are functionalized to enhance the electrode–electrolyte contact, ion diffusion, charge accumulation, and redox reactions. Our practical approach utilizes date palm fronds to produce cost-effective, eco-friendly, and flexible activated carbon electrodes with hierarchical porous nanostructures for supercapacitor applications.

## Figures and Tables

**Table 1:** Structural properties of the activated carbon calculated from N_2_ adsorption isotherms.

	CO_2_-Activated	NaOH-Activated
BET Specific Surface Area	603.5 m^2^/g	1011 m^2^/g
DFT method summary:		
Pore volume	0.401 cm^3^/g	0.452 cm^3^/g
Surface area	611.337 m^2^/g	1047.874 m^2^/g
Lower confidence limit	1.232 nm	0.705 nm
Fitting error	0.229%	0.039%
Pore width (mode)	1.232 nm	1.41 nm
DA method summary:		
Best E	4.602 kJ/mol	17.211 kJ/mol
Best n	1.000	1.000
DA Micropore Volume	0.416 cm^3^/g	0.486 cm^3^/g
Pore Diameter (mode)	1.56 nm	1.2 nm

## Data Availability

Not applicable.
